# Quantitative Trait Loci Identify Functional Noncoding Variation in Cancer

**DOI:** 10.1371/journal.pgen.1005826

**Published:** 2016-03-03

**Authors:** Holger Heyn

**Affiliations:** Cancer Epigenetics and Biology Program (PEBC), Bellvitge Biomedical Research Institute (IDIBELL), 08908 L’Hospitalet de Llobregat, Barcelona, Catalonia, Spain; Albert Einstein College of Medicine, UNITED STATES

## Abstract

The interpretation of noncoding alterations in cancer genomes presents an unresolved problem in cancer studies. While the impact of somatic variations in protein-coding regions is widely accepted, noncoding aberrations are mostly considered as passenger events. However, with the advance of genome-wide profiling strategies, alterations outside the coding context entered the focus, and multiple examples highlight the role of gene deregulation as cancer-driving events. This review describes the implication of noncoding alterations in oncogenesis and provides a theoretical framework for the identification of causal somatic variants using quantitative trait loci (QTL) analysis. Assuming that functional noncoding alterations affect quantifiable regulatory processes, somatic QTL studies constitute a valuable strategy to pinpoint cancer gene deregulation. Eventually, the comprehensive identification and interpretation of coding and noncoding alterations will guide our future understanding of cancer biology.

Cancer is considered to be a genetic disease [1]. Herein, aberrations affecting protein-coding sequences and the perturbation of transcriptional regulation can drive the step-wise process of neoplastic transformation [2]. However, taking into account the wealth of alterations found in cancer genomes, the identification of functional variation presents a major challenge. Specifically, the interpretation of genetic aberrations located outside the coding context is a poorly resolved issue in cancer genomics studies. In this regard, noncoding germline variation crucially contributes to phenotype formation in healthy and pathologic contexts, and understanding of somatically acquired variance will further improve our knowledge of cancer biology [3].

## Noncoding Mutations in *Cis*-Elements As Cancer-Driving Events

The currently best characterized example of functional noncoding variation with implication in oncogenesis is seen in the recurrent somatic mutations in the proximal promoter region of the *TERT* (telomerase reverse transcriptase) oncogene [4,5]. Although *TERT* is not frequently mutated in cancer cells, its overexpression promotes cancer formation by impairing telomere-shortening related senescence. Consistently, mutations directly upstream of its transcription start site were associated to elevated gene expression levels, suggesting the noncoding variants actively contribute to the neoplastic transformation process. From a mechanistic point of view, the somatic alterations, frequently found in melanoma and other cancer types [6], create new binding motifs for Ets transcription factors and ternary complex factors (TCFs) within the *TERT* proximal promoter, resulting in overexpression of the gene in respective tumor samples [4,5]. Similarly, recurrent mutations in the promoter regions of *NDUFB9* in melanomas are predicted to disrupt SP1/KLF binding motifs, a mechanism that, however, requires further functional validation [7].

Additional examples of functional noncoding variation point to a general implication of *cis*-regulatory perturbations in oncogenesis. In particular, the leukemic oncogene *TAL1* is activated in T-cell acute lymphoblastic leukemia (T-ALL) by somatic mutations that favor the binding of activating transcription factor (TFs) [8]. Specifically, the alterations introduce binding sites for MYB that recruits further activators, including CBP. Intriguingly, the latter confers the acetylation of H3K27 and the formation of a super-enhancer, further amplifying the activation of *TAL1*. In addition to somatic mutations, structural variations can drive cancer gene deregulation by the positioning of strong *cis*-regulatory elements in the proximity of oncogenes. Here, seminal examples involve the hijacking of enhancers and super-enhancers in medulloblastoma (activating *GFI1* [9]), in multiple myeloma (*MYC* [10]), in acute myeloid leukemia (*EVI1* [11]), and the recently described activation of *TERT* by translocation events in neuroblastoma [12].

## Systematic Identification of Functional Noncoding Alterations

As illustrated by aforementioned examples, the intrinsic properties of the DNA sequence (such as TF binding motifs) can point to functional genetic alterations and guide the prioritization of variants for subsequent validation studies [13]. Moreover, the coordinated efforts of international consortia, such as ENCODE [14], ROADMAP [15], and BLUEPRINT [16], provided a comprehensive functional segmentation of the genome and it is this genome-wide annotation, based on histone marks, chromatin accessibility, or DNA modifications, that further guides the prioritization of alterations with likely impact on genome activity [17,18]. Several methods, including FunSeq [13], CADD [19], FATHMM-MKL [20], and GWAVA [21], were developed that integrate genetic variance with TF binding sites (TFBS), epigenetic marks or conservation scores, prioritizing alterations with putative impact on gene deregulation. In addition, SASE-hunter identifies signatures of accelerated somatic evolution (SASE) and regions with an excess of local somatic mutations, an elegant method to prioritize noncoding alteration for subsequent confirmation [22].

Contrary to aforementioned strategies, this review highlights the application of association studies that integrate molecular information, particularly gene expression data, to identify causal genetic alterations and their mechanistic implications in gene deregulatory processes [23]. Here, in addition to transcriptional activity, regulatory factors, such as epigenetic modification, can serve as a valuable resource to quantify gene regulatory defects of cancer genes [24]. Intriguingly, such molecular association studies not only provide an informative measure of functionality but also point to target genes, with putatively oncogenic implication. In this regard, this strategy is applicable to elucidate deregulation events of established cancer genes and provides a resource for putative novel disease-driving factors that have been left unidentified by prior studies focusing on exonic (or splicing donor site) variation as causal event.

The concept derives from Quantitative Trait Loci (QTL) analysis, the integration of germline polymorphic regions with genome-wide molecular information. Molecular traits, such as gene expression [25], DNA methylation [24], histone marks [26], or chromatin interactions [27], are utilized to bookmark differential activity of variant genetic sites and to guide their interpretation. A likely mechanistic scenario involves the differential binding of TFs to *cis*-regulatory elements that triggers differential expression of respective target genes, quantifiable by variant transcript abundance or altered epigenetic profiles. QTL studies are frequently used in population studies and contributed to the interpretation of natural human variation and disease susceptibility [25,28–31]. This review discusses the extension of QTL analysis for de novo variations in cancer genomes in order to identify cancer driving events in noncoding contexts. Gene expression as quantitative trait is particularly highlighted, as it is directly implicated in phenotype formation. However, DNA methylation also provides a valuable epigenetic marker trait through its stable character and the inheritable transmission throughout cancer cell divisions. Importantly, DNA methylation actively participates in gene regulatory processes but also represents a suitable proxy for transcription factor binding or chromatin configuration [32,33]. Hence, DNA methylation profiles reflect given regulatory settings at respective genetic loci and are particularly suitable for integrative analytic approaches. Consistently, DNA methylation QTL studies based on germline variation successfully guided the interpretation of disease risk loci [28,30,34].

## Somatic QTL Analysis Identified Putative Cancer-Driving Events

Supporting the value of QTL studies in detecting functional noncoding alterations, single loci approaches could be replicated using genome-wide profiling strategies. Particularly, *TERT* mutations represented the most frequent event in pan-cancer profiling strategies based on recurrence or expression QTL analysis [6,23,35]. Surprisingly, despite the use of hundreds of samples across various cancer types, the number of noncoding driver candidates identified in pan-cancer studies lags far behind expectations [6,23,35], considering the high number of alterations falling in putative regulatory regions. In fact, by integrating mutational and gene expression data across cancer types, *TERT* promoter variants represented the only association with genome-wide significance [23]. This can partly be explained by the tissue-specific nature of gene regulatory processes and gene expression, a phenomenon that can highly confound integrative analysis approaches [36]. Moreover, mutational profiles are unique to cancer types, further hindering an unbiased analysis across cancer types [2,37]. Thus, larger datasets are required to perform QTL analysis in a stratified manner, as cancer type restricted analyses are likely to be more sensitive for the identification of functional regulatory variance.

In this regard, a recent work highlighted the value of QTL studies by identifying putative noncoding driver events in chronic lymphocytic leukemia (CLL) [38]. In total, the study included 150 whole-genome sequenced samples and matched gene expression and epigenomic datasets, allowing a comprehensive cancer type specific association analysis. Remarkably, the study identified a densely mutated cluster on chromosome 9q13 that could be associated to differential expression of *PAX5*, a transcription factor with a role in B cell biology. Importantly, the *cis*-regulatory effect could be experimentally validated through chromatin conformation analyses and targeted genome-editing. It is of note that the high number of cancer samples also allowed a stratified analysis that further suggested a CLL subtype specific function of *PAX5* deregulation with putative cancer driver effects.

Restricting the analysis to previously defined TFBS, another study analyzed a total of 84 lymphoma samples for functional noncoding variants [39]. Integrating mutation and expression datasets using a probabilistic model termed xseq, the study determined recurrent somatic mutations with *cis*-regulating function. Interestingly, by combining protein-coding and *cis*-regulatory alterations, the work determined cancer genes, such as *MYC*, to be affected by both mechanisms and suggested they have complementary effects in oncogenesis.

## Challenges of Somatic QTL Studies

The systematic identification of differential gene regulation related to somatic alterations in cancer has, compared to their germline counterparts, particular requirements in terms of data resources. While common natural polymorphisms can be profiled using single nucleotide polymorphism (SNP) array technologies with subsequent imputing approaches to assess the main proportion of germline variance present in a given sample set, this strategy cannot be applied for somatic variance. To chart somatically acquired variance, more comprehensive strategies, such as whole genome sequencing, are required. Moreover, in contrast to common germline variants, the recurrence rate of functional somatic mutations is expected to be rather low [40]. Taking protein coding driver mutations as gold-standard for functional genetic alterations in cancer, frequencies lower than 5% for the majority of driver events can also be assumed for noncoding alterations. Low recurrence rates directly impact on downstream statistical analysis, as the power to determine significant associations is highly reduced compared to traditional QTL studies. Consequently, more comprehensive strategies to determine variance at a given genomic loci should be considered, for example the joint analysis of single nucleotide substitutions with structural alterations, such as small insertions/deletions (indels) or larger structural variants (SV). Moreover, functionally related alterations might be scattered, further diminishing the recurrence rate at single nucleotides. In this regard, alterations can have consistent impact on regulatory elements, although not affecting the exact same position [13]. Hence, the definition of recurrence can be widened by the simultaneous analysis of neighboring variants or functional units, such as TFBS, enhancer or promoter loci, to increase the variable frequencies that enter downstream association approaches. Moreover, the detection of somatic QTL is further hindered when using cancer samples as control set. Although not being mutated for the respective locus, gene expression of the putative target genes can be perturbed by other *cis*- or *trans*-acting cancer events. Hence, the availability of matched normal samples and the use of paired statistical tests highly increase the power to detect significant associations.

While for transcriptional analysis RNA sequencing represents the current gold standard [6,23,35], DNA methylation can be assessed using sequencing or array based technologies. Here, an increased resolution is usually accompanied by higher profiling costs. However, genome-scale approaches, such as the widely used Infinium HumanMethylation450 BeadChip (Illumina) or reduced representative bisulfite sequencing (RRBS) provide reasonable resolution by profiling approximately 0.5–2.0 million CpG sites in the genome, respectively [41,42]. Although this number only represents 2%–8% of the 26 million CpG sites genome-wide, both techniques are highly informative due to the probe design in established regulatory elements and the general high correlation between neighboring CpG sites, which enables the inference of DNA methylation levels at unmeasured loci [43].

Sequencing based techniques to profile molecular traits present further advantages by providing information about the local genetic setting and allowing the identification of allele specific variance [44,45]. Assuming that somatic alterations are generally heterozygous and affect regulatory events on the same chromosome, quantifying allele specific biases in expression or methylation provides further evidence for a genotype-controlled deregulation process [46]. However, allele specific expression or methylation analysis is limited by the presence of informative polymorphisms and thus the sequencing read length might be maximized to optimally resolve the regions of interest. Although allelic events provide important evidence about the effects of *cis*-regulatory alterations on their respective target regions, a common allelic location can only be assessed in haplotype resolved genomes [47]. Furthermore, cell heterogeneity in cancer complicates allelic interpretations, as common allelic events might occur in different subclones within the tumor mass.

## Associating Genetic Variance to Gene Expression and Epigenetic Traits

Following the identification of recurrent genetic variation in the profiled cancer cohort, putative functional relevant entities can be determined using association strategies. Optionally, recurrently mutated regions can be subset to pre-defined regulatory regions [17] or prioritized loci [48], however, these might not sufficiently mirror the *cis*-acting landscape in a given cancer type. As current epigenomic maps insufficiently reflect inter-individual variation and include potential biases introduced by in vitro conditions, limiting the analysis to previously annotated loci could exclude a substantial number of functional associations.

**Fig 1:**
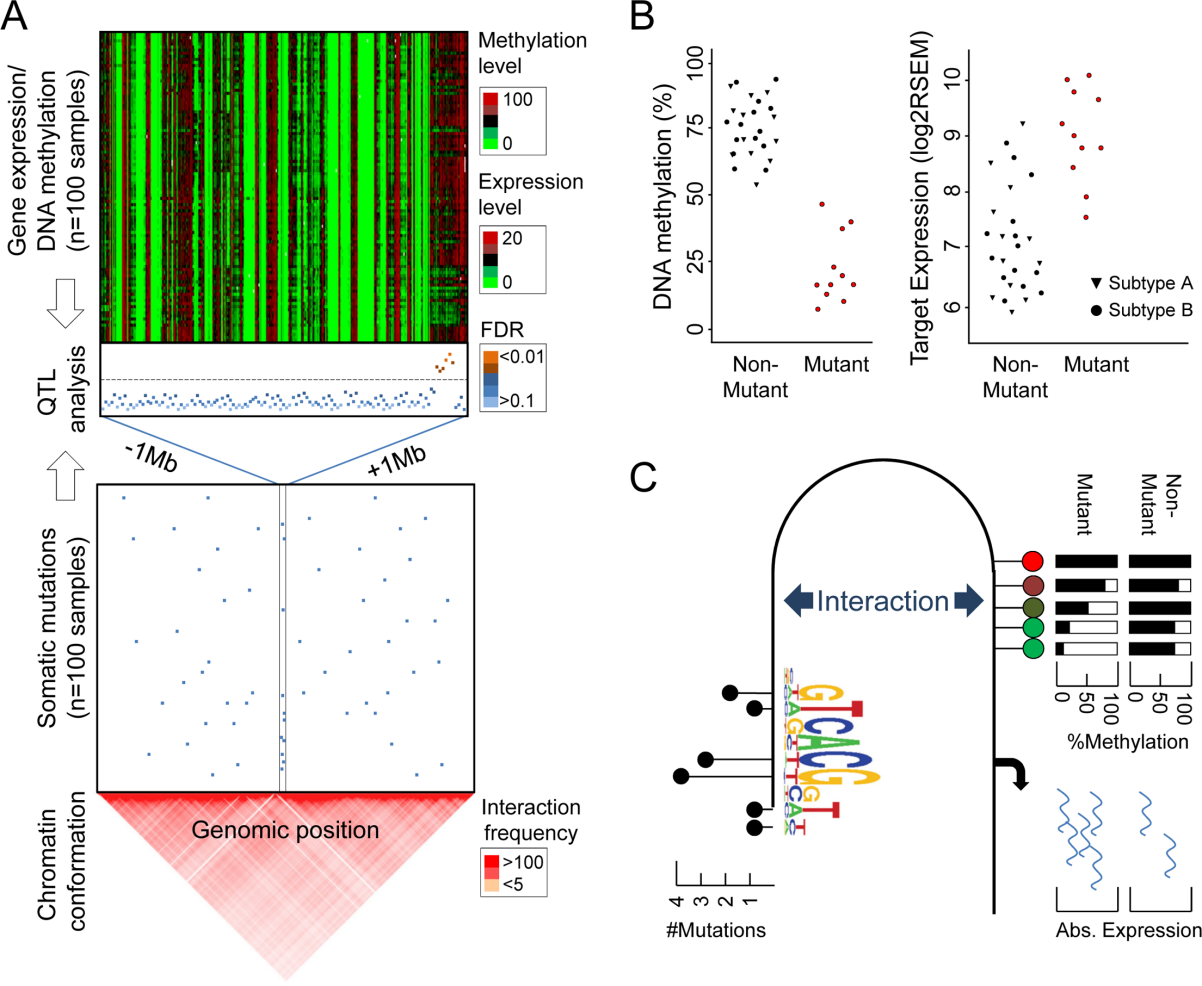
Identification of differential gene regulation associated to recurrent somatic mutations in cancer. (**A**) Framework for association studies linking genetic variance (blue dots, lower box) to gene expression or DNA methylation levels (color coded, upper box) to identify somatic Quantitative Trait Loci (QTL). Recurrent somatic variance in cancer samples is identified by whole genome sequencing, wherein different window sizes are suitable to determine frequent mutations. Statistical tests define significant *cis*-associations to gene expression or DNA methylation levels in a defined window flanking the variants (e.g., +/- 1Mb), which can be linked to additional molecular information, such as chromatin interaction frequencies in the regions of interest. (**B**) Differential DNA methylation (left) or gene expression (right) in mutant samples (red dots) point to functional somatic variation events. Stratification by cancer subtypes identifies specific events and provides further insights into the cancer type biology. (**C**) Following the identification of putative functional genetic alterations in cancer genomes, their underlying mechanisms can be elucidated through the integration of additional molecular information. Herein, the effect of mutations on the affinity of transcription factors presents valuable mechanistic insights. Moreover, spatial analysis linking variant loci to their respective target genes within the genomic space.

*Cis*-regulation on target genes is likely to be conferred by the direct physical contact of regulatory elements. Consequently, the genomic distance between the loci presents a natural barrier, with increasing distance decreasing the probability of two loci to interact. Moreover, chromosomes are organized in stable topological domains, further limiting far-reaching interaction events [49]. Consistently, interaction events between distal genomic regions and enhancer/target pairs locate predominantly within 1–2 Mb of the genome, with interaction frequency being a direct function of genomic distance [50]. Thus, limiting CpG methylation levels analysis to events flanking the recurrently mutated windows, likely captures the majority of functional *cis*-acting events (**Fig 1A**). Nevertheless, genome-wide approaches and herein the identification of *trans*-acting mechanisms, provides substantial additional information. Specifically, alterations of noncoding RNAs that control gene expression in *trans*, could provide important clues of gene deregulation events over large distances or inter-chromosomally [51]. However, genome-wide approaches are facing restrictions due to multiple hypothesis testing, which can be an important limitation considering the expected low recurrence rate of somatically acquired variance in cancer.

Several statistical approaches are suitable for the integration of genotype with gene expression or epigenetic datasets and knowledge drawn from germline QTL studies provides an informative basis and suitable guidance. Commonly applied methodologies for genomic data integration are Random Forest Selection Frequency (RFSF) based approaches, determining significant associations by repeated hierarchical clustering. RFSF was suggested to perform superior in the assessment of eQTL compared to other methods [52] and was previously applied in meQTL analysis [28,34]. An alternative to RFSF is represented by linear regression models, which are adjustable for covariates, an important issue in association studies [31]. Herein, in addition to technical variates, clinical parameters, such as tumor stage or patient age, are considerable parameters, which segregate with genetic features, such as mutation load. Additionally, regression analyses are adjustable for hidden covariates, assessable by algorithms, such as PEER [53]. While RFSF and regression models are suitable methods to detect subtle associations or those with high internal variance, respectively, more robust methods, such as correlation or hypothesis tests represent suitable alternatives and are widely used in molecular association studies. Noteworthy, a number of published tools implemented the integration of genetic variance, including somatic mutations, with gene expression data. Particularly, OncoCis [54] and FunSeq2 [13,55] combine genetic, epigenetic, and gene expression information for the detection of functional noncoding variance in cancer genomes that can be prioritized in subsequent validation studies.

The theoretical framework for the identification of causal somatic variants using QTL analysis is summarized in **Fig 1**. Following the identification of genetic variance, association methods determine significant relationships to gene expression or DNA methylation levels. These putative *cis*-regulatory loci are prioritized for subsequent characterization of underlying mechanisms through the integration of further regulatory mechanisms, such as TF binding or chromatin conformation.

## Conclusion

Considering the wealth of alterations found in cancer genomes, the discrimination between active and silent variants represents a critical first step to identify oncogenic genetic variation. In this regard, the integration of somatic alterations with molecular data presents a powerful approach to determine functional alterations. Particularly, regulatory quantitative trait loci analysis is suitable to define regions putatively implicated in oncogenesis; however, association analyses are adjustable to various types of molecular information. Although this review highlights the analysis of gene expression and DNA methylation as suitable markers for regulatory activity, the approach is readily adjustable to other traits, such as different epigenetic markers or even cellular phenotypes. Herein, the identification of functional alterations highly benefits from the combination of comprehensive high-resolution profiling strategies. This has to be taken into account in the design of future cancer genome studies, wherein the sole assessment of genetic variation can impede a systematic downstream analysis and let important disease-driving events remain unidentified.
